# Glucagon‐like peptide 1 infusions overcome anabolic resistance to feeding in older human muscle

**DOI:** 10.1111/acel.13202

**Published:** 2020-08-03

**Authors:** Haitham Abdulla, Bethan E. Phillips, Daniel J. Wilkinson, Marie Limb, Tereza Jandova, Joseph J. Bass, Debbie Rankin, Jessica Cegielski, Mariwan Sayda, Hannah Crossland, John P. Williams, Kenneth Smith, Iskandar Idris, Philip J. Atherton

**Affiliations:** ^1^ MRC‐Versus Arthritis Centre for Musculoskeletal Ageing Research Clinical, Metabolic and Molecular Physiology Royal Derby Hospital Centre University of Nottingham Derby UK; ^2^ NIHR Nottingham BRC University of Nottingham Nottingham UK; ^3^ Diabetes and Endocrinology Centre University Hospitals Birmingham NHS Foundation Trust Heartlands Hospital Birmingham UK; ^4^ Department of Anaesthesia University Hospitals Derby and Burton NHS Foundation Trust Derby UK; ^5^ Department of Endocrinology and Diabetes University Hospitals Derby and Burton NHS Foundation Trust Derby UK

**Keywords:** glucagon‐like peptide 1, microvascular blood flow, muscle protein breakdown, muscle protein synthesis

## Abstract

**Background:**

Despite its known insulin‐independent effects, glucagon‐like peptide‐1 (GLP‐1) role in muscle protein turnover has not been explored under fed‐state conditions or in the context of older age, when declines in insulin sensitivity and protein anabolism, as well as losses of muscle mass and function, occur.

**Methods:**

Eight older‐aged men (71 ± 1 year, mean ± *SEM*) were studied in a crossover trial. Baseline measures were taken over 3 hr, prior to a 3 hr postprandial insulin (~30 mIU ml^−1^) and glucose (7–7.5 mM) clamp, alongside I.V. infusions of octreotide and Vamin 14 (±infusions of GLP‐1). Four muscle biopsies were taken, and muscle protein turnover was quantified via incorporation of ^13^C_6_ phenylalanine and arteriovenous balance kinetics, using mass spectrometry. Leg macro‐ and microvascular flow was assessed via ultrasound and anabolic signalling by immunoblotting. GLP‐1 and insulin were measured by ELISA.

**Results:**

GLP‐1 augmented muscle protein synthesis (MPS; fasted: 0.058 ± 0.004% hr^−1^ vs. postprandial: 0.102 ± 0.005% hr^−1^, *p* < 0.01), in comparison with non‐GLP‐1 trials. Muscle protein breakdown (MPB) was reduced throughout clamp period, while net protein balance across the leg became positive in both groups. Total femoral leg blood flow was unchanged by the clamp; however, muscle microvascular blood flow (MBF) was significantly elevated in both groups, and to a significantly greater extent in the GLP‐1 group (MBF: 5 ± 2 vs. 1.9 ± 1 fold change +GLP‐1 and −GLP‐1, respectively, *p* < 0.01). Activation of the Akt‐mTOR signalling was similar across both trials.

**Conclusion:**

GLP‐1 infusion markedly enhanced postprandial microvascular perfusion and further stimulated muscle protein metabolism, primarily through increased MPS, during a postprandial insulin hyperaminoacidaemic clamp.

## Graphical abstract

Muscle myofibrillar protein synthesis rises following GLP‐1 infusion during postprandial clamp

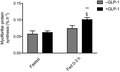

**Abbreviations**
CEUS: contrast‐enhanced ultrasound
DPP‐4: dipeptidyl peptidase‐4
EAAs: essential amino acids
FSR: fractional synthesis rate
GLP‐1: glucagon‐like peptide 1
LBF: leg blood flow
MBV: microvascular blood volume
MFV: microvascular flow velocity
MPB: muscle protein breakdown
MPS: muscle protein synthesis
MVR: microvascular recruitment

## 1 INTRODUCTION

Increases in muscle protein synthesis (MPS) and decreases in breakdown (MPB) after feeding, which recoup muscle protein losses during fasting, are crucial for muscle maintenance. The primary driver of increased MPS are dietary essential amino acids (EAAs) (Atherton et al., 2010; Cuthbertson et al., 2005), which induce a marked (~2‐ to 3‐fold), but transient (~2‐3 hr) elevation in MPS, even in the presence of continued EAA availability (Atherton et al., 2010). Conversely, insulin promotes muscle protein anabolism, primarily via suppression of MPB (Abdulla, Smith, Atherton, & Idris, 2016; Cuthbertson et al., 2005), concomitantly enhancing microvascular recruitment, increasing substrate delivery to muscle (Barrett, Wang, Upchurch, & Liu, 2011) and promoting the uptake and storage of glucose in muscle cells (Timmerman et al., 2010).

In ageing, skeletal muscle becomes resistant to the anabolic effects of dietary EAA and insulin—termed ‘anabolic resistance’ (Cuthbertson et al., 2005; Moore et al., 2015; Wall et al., 2015). This manifests as an inability of muscle to sequester EAA during the postprandial state (Cuthbertson et al., 2005; Katsanos, Kobayashi, Sheffield‐Moore, Aarsland, & Wolfe, 2005), while the anti‐catabolic effect of insulin in reducing MPB is also impaired (Wilkes et al., 2009) (as is the inhibition of whole‐body protein breakdown; Guillet et al., 2004)—ostensibly leading to net losses of muscle protein over time (Cuthbertson et al., 2005; Moore et al., 2015). In addition, glucose uptake and storage in muscle are also reduced, as a result of impaired insulin signalling (Castellino, Luzi, Simonson, Haymond, & DeFronzo, 1987; Churchward‐Venne et al., 2012; Cuthbertson et al., 2005; Hamer et al., 2013; Moore et al., 2015; Wilkes et al., 2009). Further, there is a loss of vascular function and nutrient‐induced blood flow with age (Keske et al., 2016), which may contribute further to dysregulated substrate delivery and impaired glycaemic control (Barzilay et al., 2009; Fujita, Glynn, Timmerman, Rasmussen, & Volpi, 2009).

A major hormonal response to nutrition (in addition to insulin) is the release of gut‐derived hormones. In particular, the incretin hormone, glucagon‐like peptide (GLP‐1), has emerged as a key regulator of postprandial glucose metabolism through its ubiquitously expressed receptor (GLP‐1r) (Delgado et al., 1995). GLP‐1 is released from the neuroendocrine L cells of the gut in response to intake of macro‐nutrients (Elliott et al., 1993), limiting postprandial hyperglycaemia via enhancing β‐cell insulin secretion, suppressing α‐cell glucagon and delaying gastric emptying (Holst, Vilsbøll, & Deacon, 2009; Kreymann, Williams, Ghatei, & Bloom, 1987; Willms et al., 1996). Previous data also demonstrate that GLP‐1 stimulates muscle glucose uptake (Idris, Patiag, Gray, & Donnelly, 2002), likely through enhancing skeletal muscle microvascular blood flow (MBF) in an insulin‐independent manner (Subaran et al., 2014).

Crucially, recent data have emerged suggesting a novel role of GLP‐1 in relation to muscle protein metabolism and muscle mass regulation. For example, longer‐term observational studies with inhibitors of the GLP‐1‐degrading enzyme, dipeptidyl peptidase‐4 (DPP‐4), demonstrate improved muscle mass preservation in older age (Bouchi et al., 2018). Moreover, recently a comprehensive pre‐clinical study demonstrated that exendin‐4—a peptide agonist of GLP‐1r—suppressed the expression of molecules involved in muscle atrophy, for example atrogin 1 and MURF‐1, and preserved muscle in mouse models of muscle wasting (Hong, Lee, Jeong, Choi, & Jun, 2019). However, evidence of a direct effect of GLP‐1 on human muscle protein metabolism is lacking and its potential impact on ‘anabolic resistance’ in ageing muscle is unexplored. Therefore, the aims of this study were to determine the acute impact of GLP‐1 in relation to muscle protein metabolism. We hypothesised GLP‐1 would promote greater protein anabolism in older muscle in response to amino acid feeding during a postprandial insulin clamp.

## 2 RESULTS

### 2.1 Subject characteristics

The physical and demographic characteristics of study participants are described in Table 1.

**TABLE 1:** Characteristics of study participants

Parameter	Participants (n = 8)
Age (year)	71 ± 1
Height (m)	1.77 ± 0.03
Weight (kg)	83 ± 4
BMI (kg m^−2^)	26.2 ± 0.6
Leg muscle mass (g)	9201 ± 28
Sarcopenic index (kg m^−2^)	8.13 ± 0.18
Fasting plasma glucose (mM L^−1^)	5.6 ± 0.2
Ethnicity	Caucasian: 7 (87.5%) South Asian: 1 (12.5%)

Note

Data are presented as mean ± *SEM*.

### 2.2 GLP‐1 and Insulin concentrations

**FIGURE 1:**
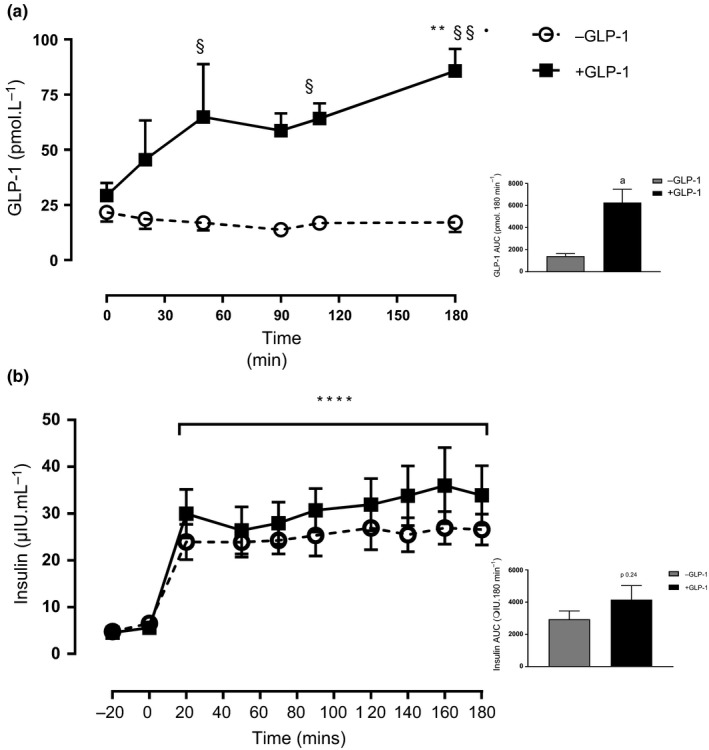
GLP‐1 concentration over 180 min postprandial clamp (a) and AUC above baseline (inset). Insulin concentrations at fasted and following fed‐state clamp with and without GLP‐1 (b) and insulin AUC above baseline (inset). **p < 0.01 versus respective fasted value, ^§^ *p* < 0.05 versus – GLP‐1 respective time point, ^§§^ *p* < 0.01 versus –GLP‐1 respective time point, ****p < 0.0001 versus respective fasted values. ^a^ *p* < 0.01 versus – GLP‐1. Data reported as mean ± *SEM*

GLP‐1 levels at baseline in the two were similar; however, upon infusion of GLP‐1, levels rose rapidly peaking around 40 min, but remained unchanged throughout, in the control group. Mean GLP‐1 concentration over the postprandial period was 63 ± 15 pmol L^−1^ and 17 ± 4 pmol L^−1^ with and without GLP‐1 infusion, respectively, and the AUC above baseline was significantly greater in the GLP‐1 group (Figure 1a) (inset). Postabsorptive insulin concentrations were similar at baseline in both groups (5.1 ± 0.5 and 5.6 ± 0.9 μIU ml^−1^ with and without GLP‐1, respectively). During the postprandial clamp with insulin alone, levels rose to 25 ± 0.4 μIU ml^−1^ and to 31 ± 1.3 μIU ml^−1^ when GLP‐1 was co‐infused (*p* < 0.001 vs. fasting for both) (Figure 1b); AUC above baseline is shown in the inset, for −GLP‐1 and +GLP‐1, *p* = 0.24.

### 2.3 Phenylalanine concentration and enrichment

**FIGURE 2:**
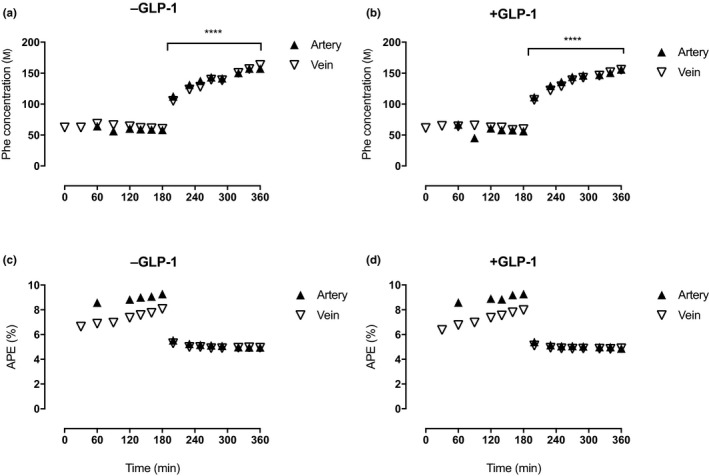
Phenylalanine concentrations (A&B) and enrichment (C&D) at baseline (fasted state) and following postprandial clamp (fed state), with and without GLP‐1. Steady‐state enrichment (A&B) and concentrations in fasted and fed state (C&D) in the femoral artery and vein with and without GLP‐1. ***p < 0.001 versus baseline, ****p < 0.0001 versus baseline

Arterial phenylalanine concentrations were similar at baseline in both groups (58.5 ± 2.7 μM −GLP‐1 vs. 60.8 ± 3.4 μM +GLP‐1 and rose significantly over the first 90 min of the postprandial period in both groups more than doubling to 135.8 ± 6.8 μM and 130.5 ± 2.7 μM, respectively, both *p* < 0.0001 Figure 2a,b) and remained elevated throughout. Phenylalanine concentrations were used as a proxy to illustrate the effect of Vamin infusion on circulating AA concentrations, mimicking a feeding response.

**FIGURE 3:**
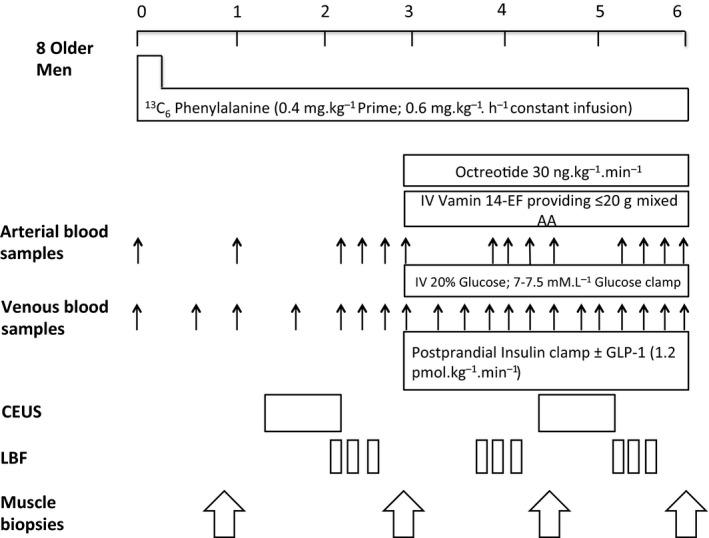
Schematic representation of study protocol. 8 older men studied in a crossover design in the fasted + fed state, with and without GLP‐1. CEUS, contrast‐enhanced ultrasound; LBF, leg blood flow

Phenylalanine enrichment reached a steady state rapidly during the baseline period and then fell similarly in both groups to a new steady state during the postprandial clamp (Figure 2c,d), due to the presence of unlabelled Phe in the Vamin infusion. Phenylalanine kinetics were determined over three periods as outlined below, when leg blood flow was simultaneously measured (see protocol Figure 3).

### 2.4 Total leg blood flow and microvascular blood flow

**FIGURE 4:**
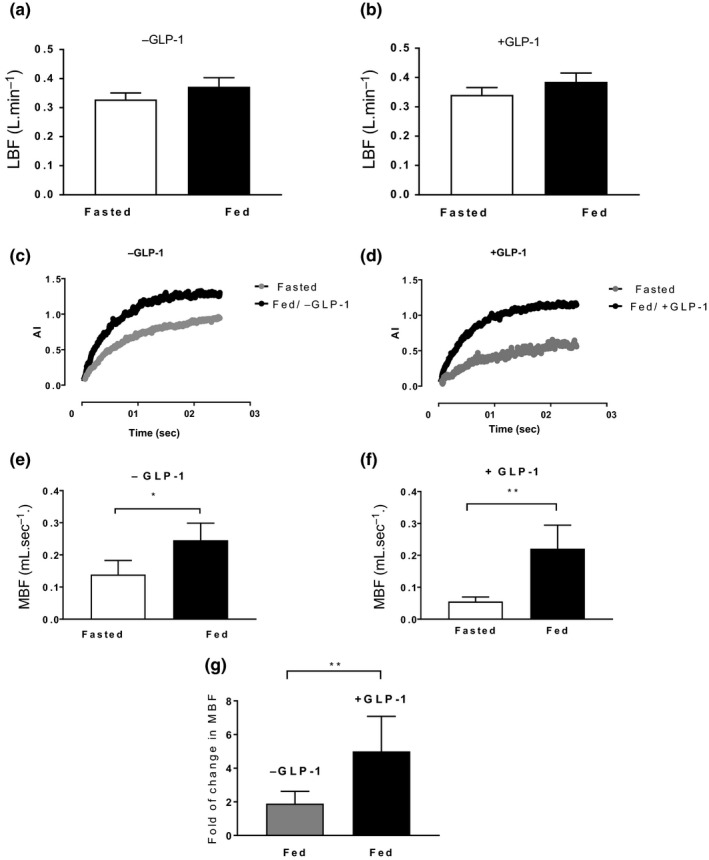
Microvascular responses to postprandial insulin ± GLP‐1 measured by CEUS. LBF measured by Doppler ultrasound at baseline and during the postprandial clamp with and without GLP‐1 is shown in b and a, respectively. Graphs c and d show acoustic intensity (AI) generated from microvascular microbubble contrast refilling (corresponding to MBV) plotted against time. MBF at baseline and following feeding alone and with GLP‐1 is shown in e and f, respectively. Fold change in MBF from baseline following feeding with and without GLP‐1 is shown in g. *p < 0.05, **p < 0.01. AI, acoustic intensity; MBF, microvascular blood flow; MBV, microvascular blood volume; LBF, leg blood flow. Data presented as mean ± *SEM*

Although total leg blood flow rose slightly, during the postprandial clamp in both groups, this was not significant (0.34 ± 0.02 vs. 0.39 ± 0.03 L min^−1^ −GLP‐1, and 0.33 ± 0.02 vs. 0.37 ± 0.031 L min^−1^, +GLP‐1, Figure 4a,b). With regard to microvascular recruitment, replenishment curves of AI at baseline and during the postprandial clamp, with and without GLP‐1, are shown in Figure 4c,d, showing the impact of the postprandial clamp on tissue perfusion. In response to the clamp without GLP‐1, MBF increased from 0.14 ± 0.04 to 0.25 ± 0.05 ml s^−1^ (baseline vs. postprandial clamp, *p* < 0.05, Figure 4e), whereas co‐infusion of GLP‐1 increased MBF from 0.06 ± 0.01 to 0.22 ± 0.07 ml s^−1^ (baseline vs. postprandial clamp, *p* < 0.01, Figure 4f), representing a fold of change from baseline of 5 ± 2.1 (+GLP‐1) versus 1.9 ± 0.7 (−GLP‐1), *p* < 0.01, Figure 4g.

### 2.5 Myofibrillar Protein Synthesis (MPS)

**FIGURE 5:**
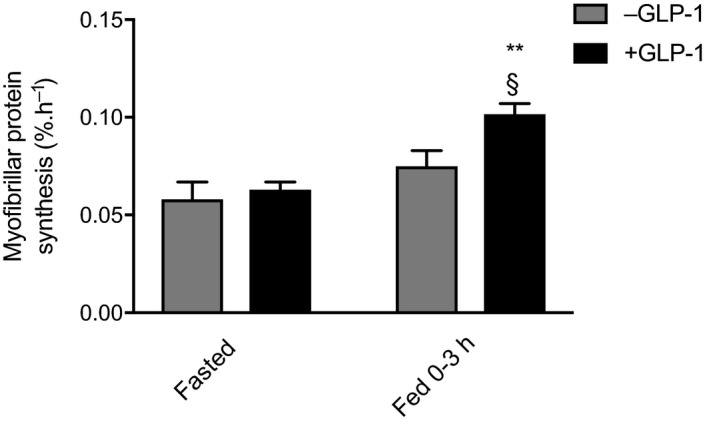
Fasted versus fed myofibrillar muscle protein synthesis under postprandial clamp conditions, with and without GLP‐1. **p < 0.01 versus respective fasted, §*p* < 0.05 versus fed −GLP‐1. FSR, fractional synthesis rate. Data reported as means ± *SEM*

MPS was similar in both experiments under postabsorptive conditions (0.058 ± 0.008% hr^−1^ vs. 0.063 ± 0.003% hr^−1^, −GLP‐1 vs. +GLP‐1, respectively, Figure 5). During the postprandial clamp, MPS rose slightly, but non‐significantly in the −GLP‐1 group (0.075 ± 0.008% hr^−1^, *p* = 0.23); however, co‐infusion of GLP‐1 resulted in a significantly greater rise in MPS (0.102 ± 0.005% hr^−1^, *p* < 0.01, also *p* < 0.05 compared to MPS of −GLP‐1 in postprandial phase).

### 2.6 Phenylalanine leg turnover kinetics

**FIGURE 6:**
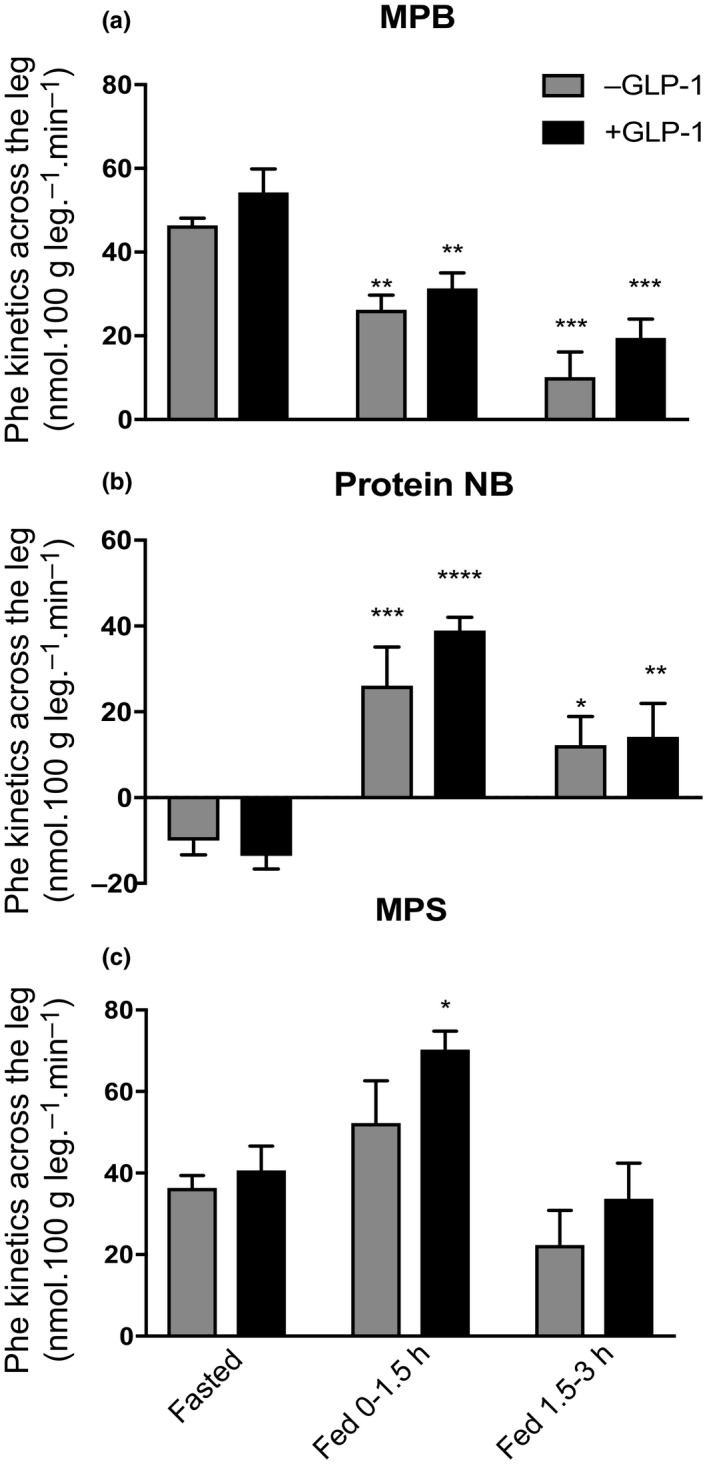
Phenylalanine kinetics across the leg in response to postprandial insulin with and without GLP‐1 in a: MPB, b: protein NB and c: MPS. PP INS, postprandial insulin; MPB, muscle protein breakdown; NB, net balance. *p < 0.05 versus respective fasted, **p < 0.01 versus respective fasted, ***p < 0.001 versus respective fasted, ****p < 0.0001 versus respective fasted. Data reported as means ± *SEM*

Phenylalanine kinetics across the leg are summarised in Figure 6. Phenylalanine delivery to the leg at baseline was not different in both groups and increased during the postprandial clamp (272 ± 23 vs. 752 ± 48 nmol 100 g leg^−1^ min^−1^, and 244 ± 20 vs. 690 ± 45 nmol 100 g leg^−1^ min^−1^, both *p* < 0.001, with and without GLP‐1, respectively).

The rate of appearance, Ra, of phenylalanine was similar in both groups at baseline (46 ± 2 nmol 100 g leg^−1^ min^−1^, −GLP‐1, and 54 ± 2 nmol 100 g leg^−1^ min^−^1, +GLP‐1) during the postprandial clamp Ra fell similarly, at 0–90 min and 90–180 min in both −GLP‐1, (to 26 ± 2 and 10 ± 6 nmol 100 g leg^−1^ min^−1^, −43%, *p* < 0.005 and −78%, *p* < 0.001, respectively) and +GLP‐1 groups, (to 31 ± 4 and 20 ± 5 nmol 100 g leg^−1^ min^−1^, −43% days, *p* < 0.01 and −63%, *p* < 0.001, respectively; Figure 6a).

Muscle protein net balance (NB) was negative in both groups in the fasted state (−10.0 ± 3 vs. −13.6 ± 3 nmol 100 g leg^−1^ min^−1^, −GLP‐1 and +GLP‐1, respectively). NB became significantly more positive during postprandial clamp at both 0–90 and 90–180 min (−GLP‐1, 26.1 ± 9 nmol 100 g leg^−1^ min^−1^, *p* < 0.001 and 12.2 ± 7 nmol 100 g leg^−1^ min^−1^, *p* < 0.05. +GLP‐1 39.0 ± 3 nmol 100 g leg^−1^ min^−1^, *p* < 0.001 and 14.2 ± 8 nmol 100 g leg^−1^ min^−1^, *p* < 0.01). The difference between the two groups (−GLP‐1 vs. +GLP‐1) was not significant at both stages of feeding (Figure 6b).

The rate of disappearance (Rd), that is synthesis, was not different between the two in the fasted state (36.4 ± 3 vs. 40.6 ± 6 nmol 100 g leg^−1^ min^−1^). The Rd rose slightly, but not significantly, in the postprandial state without GLP‐1 (52.3 ± 10 nmol 100 g leg^−1^ min^−1^); however, during GLP‐1 infusion, Rd was significantly elevated during the 0‐90 min (70.3 ± 5 nmol 100 g leg^−1^ min^−1^, *p* < 0.05), before returning to fasted levels at 90–180 min in both groups (Figure 6c).

### 2.7 Anabolic signalling

Phosphorylation of both AKT Ser^473^ and p70 S6K Thr^389^ rose significantly in response to the insulin and AA clamp (2.5‐ and 5.7‐fold increment from fasted, respectively), with no additional effect observed with GLP‐1 infusion (3.1‐ and 5.3‐fold increment from fasted, respectively) (Figure S1: B and E). Neither mTOR Ser^2448^, eEF2 Thr^56^, 4E‐BP1 Thr^37/46^ nor TSC2 Thr^1462^ activities were affected by the postprandial clamp (Figure S1: A, C, D and F).

### 2.8 Gene expression of GLP1r

Our data show sufficient expression of the receptor in both skeletal muscle cells and skeletal muscle tissue. Data in Figure S2 show gene expression of *GLP1r* in different human tissue/cell types expressed as fold difference in relation to the housekeeping gene (RPL13A).

## 3 DISCUSSION

In the present study, we demonstrate an anabolic effect of GLP‐1 in skeletal muscle of older humans under fed‐state conditions. In line with previous findings in older individuals, MPS was resistant to the impact of intravenous AA feeding under postprandial insulin conditions (in the absence of GLP‐1) (Cuthbertson et al., 2005; Mitchell et al., 2017). In contrast, GLP‐1 infusions adjuvant to the intravenous AA/insulin infusions resulted in a significant augmentation of MPS over −GLP‐1 conditions, as such rescuing anabolic responses to AA feeding. Finally, a sustained suppression of MPB during insulin clamps was observed throughout the postprandial insulin clamp, and to the same extent in both groups, indicating that GLP‐1 had no added effect upon the inhibition of MPB.

Impaired anabolic responses to nutrition are a hallmark of ageing muscle (Cuthbertson et al., 2005; Wall et al., 2015). Here, we demonstrate for the first time through *both* direct incorporation and A‐V balance methods that GLP‐1 infusion under postprandial insulin, hyperaminoacidaemic clamp conditions, further enhanced fed‐state MPS rates in humans, possibly indicating a direct effect of GLP‐1 at a cellular level in muscle. We also detected GLP‐1r expression in muscle; in line with this, other workers have shown that GLP‐1r agonists can induce protein kinase B (Akt), mitogen‐activated protein kinases and p70 ribosomal S6 kinase (p70S6K) signalling in skeletal muscle cells (González, Acitores, Sancho, Valverde, & Villanueva‐Peñacarrillo, 2005; Villanueva‐Peñacarrillo et al., 2011). Since these pathways are involved in anabolic responses to nutrition driven by AA (Fujita et al., 2007) and insulin (Hillier, Long, Jahn, Wei, & Barrett, 2000), we investigated the activation of key signalling molecules in the absence and presence of GLP‐1. However, despite enhanced postprandial MPS with GLP‐1 infusions, and increased phosphorylation of, for example, AKT Ser^473^ and p70 S6K Thr^389^ during the clamps, there were no additional differences apparent with GLP‐1 that could explain the elevated MPS. Similarly, mTOR Ser^2448^, eEF2 Thr^56^, 4E‐BP1 Thr^37/46^ and TSC2 Thr^1462^ were unaffected by the postprandial clamp and were not different between the groups. These findings further highlight the temporal and quantitative disconnect between the activity of anabolic signalling and the quantitative stimulation of MPS, in humans under fed‐state conditions (Greenhaff et al., 2008), and further work is needed to define these limitations. On long‐term provision of therapeutic GLP‐1r agonists, mainly to achieve weight loss and optimise glucose control, the resultant weight loss was predominantly fat mass and to a lesser extent some lean body mass loss has been reported (Sargeant et al., 2019). Nonetheless, acutely, GLP‐1 infusions have clear translational potential in muscle mass attainment in settings where intentional weight loss is not the primary outcome being sought.

In order to seek other mechanisms relating to GLP‐1's effects, we also quantified muscle perfusion. Age‐related attenuation of vascular responses to feeding is well documented, and we have previously described the existence of fed‐state microvascular resistance in older age and its co‐existence with impaired muscle anabolic response (Phillips et al., 2014). We demonstrate in the present study a greater increase in muscle MBF in response to feeding alongside GLP‐1 infusion (vs. −GLP‐1). Although some argue that increased tissue perfusion is a driver of enhanced protein anabolism, for example increases in MPS (Timmerman et al., 2010), our previous studies investigating the impact of altering blood flow pharmacologically (Phillips et al., 2014) and muscle microvascular perfusion following resistance exercise training (Phillips et al., 2012) demonstrated no additional anabolic effects in relation to muscle protein turnover. Notably, in our study, GLP‐1 was infused intra‐arterially in contrast to studies in rats (Chai et al., 2012) and humans (Sjøberg, Holst, Rattigan, Richter, & Kiens, 2014; Subaran et al., 2014), where GLP‐1 was infused intravenously. Given the short half‐life of GLP‐1, the marked effects we observe on MBF were possibly due to the direct vasodilatory properties of GLP‐1. Finally, enhanced MBF may instead play a crucial role in muscle glucose uptake (Vincent et al., 2004).

Recently, chronic provision of antiglycaemic DPP‐4 inhibitors was shown to result in increases in muscle mass (Bouchi et al., 2018), although the mechanistic basis for the observed benefits was not studied. Similarly, recent work in mice has shown the potential of GLP‐1 agonism in relation to skeletal muscle maintenance in catabolic disease models (Hong et al., 2019). Our data indicate that GLP‐1 infusion restores the anabolic response to EAA provision in older muscle, which may help explain the increase or maintenance in muscle mass observed in these previous studies. In the context of diabetes, GLP‐1 therapy is widely used in clinical practice, to improve glucose uptake and disposal, and given that muscle loss and the incidence of sarcopenia is accelerated in patients with diabetes (Park et al., 2007), GLP‐1 therapies may also have a role to play in maintaining muscle mass.

Our study is limited by the nature of its short duration in demonstrating these metabolic and microvascular gains. As we have previously reported significant sexual dimorphism in MPS in response to mixed meal ingestion on older men and women (Smith et al., 2008), we studied only older men, an established model of anabolic resistance, to ensure minimal confounding factors (i.e. mixed sex) in testing the effect of GLP‐1 infusion on older human muscle fed‐state MPS. Therefore, our findings would need to be confirmed in older women and indeed in other populations with metabolic disease. Nonetheless, for the first time in ageing human muscle, we are able to demonstrate that GLP‐1 infusions could overcome anabolic resistance to feeding and enhance muscle MBF. Following these findings, further mechanistic studies and clinical trials are required to elucidate the impact of the broad array of GLP‐1 therapies available (agonists/DDP‐4 inhibitors, etc.) in relation to muscle maintenance.

## 4 MATERIALS AND METHODS

### 4.1 Subjects and design

The study was approved by The University of Nottingham Ethics Committee (Reference Number: G12122013 MSGEM) and was conducted in line with the Declaration of Helsinki, 2013. Eight healthy male volunteers (65–75 years of age, see Table 1 for subject characteristics) from the local area were recruited into the study, via mailshot. A comprehensive clinical examination and metabolic screening were conducted at the Royal Derby Medical School, Derby. Subjects with metabolic disease, lower limb musculoskeletal abnormalities, acute cerebrovascular or cardiovascular disease, active malignancy, uncontrolled hypertension, and BMI <18 or >28 kg m^2^, on medications that impact muscle protein metabolism or modulate vascular tone or have known allergy to any of the study drugs, were excluded. All volunteers were studied following overnight fasting of 10–14 hr. Each volunteer was studied on two occasions, at least 3 weeks apart. Volunteers were randomly assigned to receive either glucagon‐like peptide‐1 (GLP‐1) infusion into the femoral artery in one leg or placebo in the contralateral leg. Volunteers were blinded to which visit they would receive GLP‐1.

### 4.2 Conduct of the study

#### 4.2.1 Reporting and initial preparation

On the morning of the study, volunteers reported to the Clinical Physiology Laboratory at 0800. Following a DXA scan, volunteers lay supine on a bed. Three polyethylene cannulae (20G × 2 & 18G × 1) for intravenous infusions were inserted, distributed between the two forearms. This was followed by the insertion of femoral venous and arterial cannulae into the femoral artery and vein of the leg designated for study. The area below the inguinal ligament was anaesthetised before introduction of wire‐guided femoral catheters using ultrasound scan guidance (Philips iU22 Ultrasound, Bothell, WA, USA).

#### 4.2.2 Labelled AA infusion

At time zero after baseline venous and arterial blood sampling, an infusion of L‐[ring‐^13^C_6_]‐phenylalanine (Cambridge Laboratories, MA, USA) was started (prime 0.4 mg kg^−1^. then infused at 0.6 mg kg^−1^ hr^−1^) and was continued until the end of the study (a total of 6 hr, see study protocol for timings).

#### 4.2.3 Muscle biopsies

Baseline muscle biopsies were taken at 60 min (after attainment of steady‐state isotope labelling) and 180 min following initiation of infusion and immediately before start of postprandial clamp. Two further muscle biopsies were taken at 90 min and 180 min postinitiation of the clamp.

#### 4.2.4 Postprandial clamp

Immediately following the second baseline muscle biopsy, peripheral intravenous infusions of octreotide (Novartis, Surry, UK), insulin Actrapid (Novo Nordisk, Gatwick, UK), 20% glucose (Baxter, UK) and mixed AA‐Vamin 14‐EF (Fresenius Kabi Ltd, Runcorn, UK) ± femoral arterial GLP‐1 infusion (Bachem AG, Bubendorf, Switzerland) were started. Octreotide was infused at a rate of 30 ng kg^−1^ min^−1^ (Greenhaff et al., 2008). Glucose and insulin were infused as previously described (DeFronzo, Tobin, & Andres, 1979) aiming to clamp glucose at 7–7.5 mM L^−1^ and insulin at modest postprandial levels of around 30 μIU ml^−1^. Vamin 14‐EF was started at a prime rate of 34 mg kg^−1^ followed by a constant infusion rate of 102 mg kg^−1^ hr^−1^ for 3 hr. GLP‐1 was obtained as a powder and stored at −20°C. On the day of the study, it was dissolved in 2 ml of sterile 0.9% saline, diluted and infused into the femoral artery at a constant rate of 1.2 pmol kg^−1^ min^−1^ (Subaran et al., 2014).

#### 4.2.5 Contrast‐enhanced ultrasound

At 145 min following the start of labelled AA infusion, baseline measurement of microvascular parameters was conducted using contrast‐enhanced ultrasound (CEUS—Philips iU22 Ultrasound, Bothell, WA, USA). Sonovue^TM^ (Bracco, Courcouronnes, France) was infused via a peripheral vein at an initial rate of 2 ml min^−1^ for 1 min and 1 ml min^−1^ for a further 2 min. During the three‐min duration, three cycles of flash/replenishment videos were recorded. Further assessment of microvascular parameters was made at 120 min following the start of the postprandial clamp.

#### 4.2.6 Blood sampling and leg blood flow

Regular venous and arterial blood samples were taken throughout. During the first 120–180 min, three sets of leg femoral artery blood flow (LBF) were taken. This continued during the 3‐hr postprandial clamp period; regular arterial and venous samples were taken, in addition to regular assessment of LBF (see protocol, Figure 3). Regular femoral venous samples were taken every 5–10 min during the 3‐hr postprandial period to help titrate glucose infusion to ensure blood glucose stays at postprandial levels of 7–7.5 mmol L^−1^.

At the end of the study, participants were monitored and fed before being allowed to leave. Volunteers returned after at least 3 weeks for the crossover study on the contralateral leg.

### 4.3 Anthropometric indices

Body mass index (BMI) was defined as weight in kg height in m^−2^. Sarcopenic index corresponds to the appendicular skeletal muscle mass index (ASMI), which was calculated as appendicular skeletal muscle (ASM) in kg height in m^−2^.

### 4.4 Laboratory analysis and measurements

#### 4.4.1 Plasma insulin and GLP‐1 concentrations

Commercial ELISA kits (Milliplex Map kit, EMS Millipore, Germany) were used to determine insulin, C peptide and GLP‐1 concentrations. For GLP‐1, blood was collected in P800 tubes, to stabilise GLP‐1. We used the Milliplex Map Kit—Human Metabolic Hormone Magnetic Bead Panel (Cat #HMHEMAG‐34K) with Luminex MAGPIX detection for determination of GLP‐1 (validated range 2.7–2000 mg/ml) and GIP (glucose‐dependent insulinotropic peptide) (validated range 1.4–1000 pg/ml), recovery for both was 103% from serum.

#### 4.4.2 Micro‐ and macrovascular blood flow parameters

Two video recordings were obtained (baseline and postintervention) by the end of each study. Video data were then exported to the quantification software (QLab, Philips, Andover, MA, USA) for analysis. Regions of interest were drawn avoiding areas of connective tissue and large vessels and copied into each file to ensure that regions were identical for each recording. The period immediately following flash (0.57 s) was used to calculate the background acoustic intensity (AI), an arbitrary unit, attributable to rapidly filing larger non‐exchange vessels and tissue echogenicity. The mean AI during this was calculated and subtracted from all subsequent values during that replenishment period. Then, the mean AI across all three flash/replenishment recording cycles was calculated after background correction and was curtailed at 24 s. The software automatically carries out calculations as described previously (Sjøberg, Rattigan, Hiscock, Richter, & Kiens, 2011; Wei et al., 1998) where AI versus time curves were fitted to the exponential function: y = A[1 –e−^β(t − Bt)^], where *t* is time in seconds, *Bt* is the time used for background subtraction, y is the AI at any given t, A is the plateau AI defined as MBV in ml in the ROI, and β is the flow rate constant (1itres s^−1^) that determines the rate of rise of AI and corresponds to the mean blood flow velocity or perfusion rate in ml per sec. of all vessels in the ROI. Then, the blood flow in ml per sec. can be defined as blood volume ⋅ blood flow velocity (*A* **⋅ **β) (Weber et al., 2006). Leg blood flow (LBF) was calculated using measurements obtained from ultrasound Doppler scan where LBF is determined from femoral artery dimensions **⋅** blood flow velocity, from the average of at least 3 measurements. Fold change from baseline was calculated as the difference between postprandial blood flow and the postabsorptive blood flow relative to postabsorptive blood flow. Net incremental area under the response curve (AUC) was calculated for each individual separately and presented as grouped analysis.

#### 4.4.3 Plasma AA concentration and Phe enrichment

For the measurement of AA concentration, internal standards were added to plasma samples, before the addition of urease solution and incubation at room temperature for 20 min. Samples were then de‐proteinised with ice‐cold ethanol for 20 min at 4°C, before centrifugation at 13,000 *g*. The supernatant containing plasma free AA was then decanted and evaporated at 90°C under N_2_. Dried AA were solubilised in 0.5 M HCl and lipids extracted in ethyl acetate, before being evaporated to dryness. AA were derivatised through the addition of equal volumes of acetonitrile (ACN) and N‐tert‐butyldimethylsilyl‐N‐methyltrifluoroacetamide (MTBSTFA) and heated to 90°C for 60 min to create tert‐butyldimethylsilyl (t‐BDMS) AA esters. Samples were allowed to cool before transfer to autosampler vials. AA concentrations were quantified using standard curves of known concentrations by GC‐MS, as per our standard approach (Wilkinson et al., 2018). The labelling (atom per cent excess [APE]) of arterialised and venous [^13^C_6_]phenylalanine was determined using GC‐MS, by measuring the ratio of labelled to unlabelled Phe (m/z 240–234). Concentrations for arterial plasma phenylalanine were determined using a [^15^N] phenylalanine internal standard (m/z 235), with reference to standard curves of known concentration (Smith & Rennie, 1996).

#### 4.4.4 Determination of muscle protein‐bound and intracellular free phenylalanine enrichment

The muscle myofibrillar fraction was isolated as previously described (Atherton et al., 2010; Greenhaff et al., 2008), and L‐[ring‐^13^C_6_]‐phenylalanine incorporation into myofibrillar protein was determined by gas chromatography combustion isotope ratio mass spectrometry (GC–C‐IRMS, Delta‐plus XP, Thermo, Hemel Hampstead, UK). Separation was achieved on a 25 m · 0.25 mm · 1.0 μ‐film DB 1701 capillary column (Agilent Technologies, West Lothian, United Kingdom). Gas chromatography mass spectrometry (GC‐MS, Agilent‐5977a, California, USA) was used to determine muscle intracellular L‐[ring‐^13^C_6_]‐phenylalanine enrichment. The sarcoplasmic fraction containing the intramuscular free amino acid pool was precipitated, and the supernatant was purified by cation‐exchange chromatography, using Dowex H^+^ resin and derivatised as their t‐BDMS derivatives before measurement of phenylalanine enrichment by GC‐MS (Atherton et al., 2010; Greenhaff et al., 2008; Mitchell et al., 2015).

#### 4.4.5 Calculation of fractional synthesis rates

FSR was calculated via measurement of the increase in L‐[ring‐^13^C_6_]‐phenylalanine enrichment into myofibrillar protein between two consecutive biopsies. The calculations used the standard precursor‐product equation:

$$\text{FSR}\;[\text{expressed as \%}\;{\text{hr}}^{‐1}]=(\Delta E_{m}/E_{p}\times 1/t)\times 100$$

where Δ*E*_m_ is the change in labelling of muscle bound L‐[ring‐^13^C_6_]‐phenylalanine between 2 biopsy samples, *E*_p_ is the mean L‐[ring‐^13^C_6_] free phenylalanine precursor enrichment in the intramuscular pool, and t is the time in hours between biopsies.

#### 4.4.6 Phenylalanine kinetics across the leg

Phenylalanine delivery to the leg was calculated as: LBF × *C*_A_. Phenylalanine release from the leg was calculated as: LBF × *C*_V_. Muscle protein net balance (NB) was calculated as (*C*_A_ − *C*_V_) × LBF. The rate of appearance, that is MPB, was calculated from the dilution of tracer across the leg, as (*E*_A_/*E*_V_ − 1) × LBF × *C*_A_; the rate of disappearance of phenylalanine, that is MPS, was calculated indirectly as the sum of MPB + muscle protein net balance (NB) where NB is (*C*_A_ − *C*_V_) × LBF, where *C*_A_ and *C*_V_ are concentration of phenylalanine in the femoral artery and vein, respectively, and *E*_A_ and *E*_V_ are phenylalanine enrichment in the femoral artery and vein, respectively (Bennet, Connacher, Scrimgeour, Jung, & Rennie, 1990).

#### 4.4.7 Immunoblotting analysis of signalling proteins

Phosphorylated protein concentrations were determined by Western blotting (Cell Signalling Technology, London, UK) for eukaryotic elongation factor 2 (eEF2) Thr^56^—Catalogue No 2331; p70 ribosomal S6 kinase (p70 S6K1) Thr^389^—Catalogue No 9234; AKT Ser^473^—Catalogue No 4060; mammalian target of rapamycin (mTOR) Ser^2448^—Catalogue No 5536; eukaryotic initiation factor 4E binding protein 1 (4E‐BP1) Thr^37/46^—Catalogue No 2855; and tuberous sclerosis complex 2 (TSC2) Thr^1462^—Catalogue No 3611. Proteins were extracted from ~20 mg of crudely minced muscle in ice‐cold buffer containing 50 mmol L^−1^ Tris‐HCl, 1 mmol L^−1^ EDTA, 1 mmol L^−1^ EGTA, 10 mmol L^−1^ β‐glycerophosphate, 0.5 mmol L^−1^ sodium orthovanadate and complete protease inhibitor cocktail (Roche, West Sussex, United Kingdom). Protein concentrations were determined using a NanoDrop 2000 (Thermo Fisher Scientific). Twenty micrograms of each protein sample was loaded onto 12% sodium dodecyl sulphate–polyacrylamide gel electrophoresis gel (Criterion XT Bis‐Tris; Bio‐Rad, Hemel Hempstead, United Kingdom) for electrophoresis at 200 V for 60 min and electroblotted to polyvinylidene difluoride membranes (Bio‐Rad). After incubation for 1 hr at room temperature with 5% milk in TBS‐T (Tris‐buffered saline and 0.1% Tween‐20), membranes were incubated overnight with primary antibody against the aforementioned targets at 4°C (New England Biolabs). Membranes were then washed for 3 × 5 min with TBS‐T and incubated for 1 hr at room temperature with 1:2000 horseradish peroxidase‐conjugated anti‐rabbit secondary antibody (New England Biolabs), before further washing with TBS‐T and incubation for 5 min with chemiluminescence reagents (Immobilon Western Chemiluminescent HRP Substrate; Millipore). Blots were imaged and quantified with the use of the ChemiDoc XRS system (Bio‐Rad). Normalisation of proteins of interest was performed against Coomassie blue staining.

#### 4.4.8 Gene expression of GLP‐1 receptor (GLP‐1r)

Total RNA from human skeletal muscle (CD56+) cells, human skeletal muscle tissue, human adipose tissue and human kidney cells (*n* = 6–8 per cell type) was extracted using TRI reagent (Sigma‐Aldrich, UK), according to the manufacturer's instructions. RNA was resuspended in 20 μl RNase‐free water and quantified using a NanoDrop™ 2000 (Thermo Fisher Scientific). Reverse transcription was performed with 500 ng RNA using the High‐Capacity cDNA Synthesis Kit (Applied Biosystems, Thermo Fisher Scientific). 1 µl of cDNA (in duplicate) was added into 384‐well plates with primers specific to *GLP1R* (5′–3′; forward: GACGCTCAAGAATCCTCTGG; reverse: TCAAGAGACAGCGTGTGGTC) and SYBR Select Master Mix (Applied Biosystems, Thermo Fisher Scientific) in a total volume of 7 µl. Plates were run on a ViiA™ 7 real‐time PCR system (Thermo Fisher Scientific). The gene *RPL13A* was used for normalisation.

### 4.5 Statistical analysis

The sample size was prospectively determined based on previous local studies to detect difference in muscle metabolism. For repeated measures of MPS in the same sample, the coefficient of variation (CV) is ~3.8%. The population CVs are ~10‐12% for young and older men. For MPB, with a population CV of 15% (based on previous laboratory data) and CV of laboratory techniques also of 15% (propagated error ~21%), we were able to detect (with 80% confidence at the 5% significance level) differences in rates of MPB after feeding of ±21% (i.e. 1 *SD*). Given these parameters, the smallest number of subjects needed to detect (with 80% confidence, 5% significance level) a cross‐sectional difference between groups, or a one‐way difference on a paired basis, of 20% was 8. Analysis was conducted using Prism 7 (GraphPad, San Diego, CA). Data are presented as mean ± *SEM*. Normality of distribution was tested using D’Agostino and Pearson Omnibus normality tests. Comparison between experiments and values was made via repeated‐measures two‐way ANOVA or Student's *t* test as appropriate, with Bonferroni correction for multiple comparisons. Data were accepted as significant if *p* < 0.05.

## CONFLICT OF INTEREST

The authors declare no conflict of interest in relation to this manuscript.

## AUTHOR CONTRIBUTIONS

P.J.A, I.I, K.S, B.E.P, D.J.W and H.A conceptualised the study. H.A, J.P.W and I.I performed clinical studies. B.E.P, M.L, D.J.W, T.J, J.J.B, D.R, J.C, M.S and H.C performed laboratory sample analyses, which were revised and approved by K.S and P.J.A. H.A and B.E.P analysed microvascular data from CEUS. H.A drafted the initial manuscript, which was then reviewed and edited by I.I, K.S and P.J.A. All authors approved the final version of the manuscript.

## Data Availability

The data that support the findings of this study are available on request from the corresponding author.
