# Supplementary material for: Glucagon‐like peptide 1 infusions overcome anabolic resistance to feeding in older human muscle
Source: Aging Cell. 2020 Aug 3;19(9):e13202. doi: 10.1111/acel.13202 (PMC7511886; doi:10.1111/acel.13202)
Supplement: Supplementary file 1 [file ACEL-19-e13202-s001.docx]

**Figure S1** Coomassie blots (left panel) and change (in arbitrary units) in phosphorylation status (right panel) of A: mammalian target of rapamycin complex (mTOR), B: AKT, C: eukaryotic elongation factor 2 (eEF2), D: eukaryotic initiation factor 4E binding protein 1 (4E-BP1), E: p70 ribosomal S6 kinase (p70 S6K) and F: tuberous sclerosis complex 2 (TSC2) relative to actin in the fasted and fed state (infusion of postprandial insulin and mixed AA with and without GLP-1). ** *P* <0.01, *** *P* <0.001 vs respective fasted. Data expressed as mean ± SEM
