# Supplementary material for: Glucagon‐like peptide 1 infusions overcome anabolic resistance to feeding in older human muscle
Source: Aging Cell. 2020 Aug 3;19(9):e13202. doi: 10.1111/acel.13202 (PMC7511886; doi:10.1111/acel.13202)
Supplement: Supplementary file 2 [file ACEL-19-e13202-s002.docx]

**Figure S2**: Gene expression of *GLP1r* in different tissue/cell types. Data are presented as fold difference in relation to the housekeeping gene (*RPL13A*) for human skeletal muscle cells, human muscle tissue, human kidney cells and human adipose tissue (mean + SEM; n=6-8 per group).
